# At home monitoring of chronic adaptive deep brain stimulation for Parkinson’s disease

**DOI:** 10.1016/j.brs.2026.103028

**Published:** 2026-01-09

**Authors:** Chuyi Cui, Jin Woo Choi, Shreesh Karjagi, Kevin B. Wilkins, Aarushi Negi, Helen M. Bronte-Stewart

**Affiliations:** a Department of Neurology and Neurological Sciences, Stanford University School of Medicine, Stanford, CA, USA; b Department of Neurosurgery, Stanford University School of Medicine, Stanford, CA, USA

**Keywords:** Closed-loop DBS, Subthalamic nucleus, Local field potential, Remote digital monitoring

Dear Editor,

Exaggerated subthalamic nucleus (STN) beta oscillations and synchrony reflect pathological neural activity in Parkinson’s disease (PD) and are associated with worsening of motor symptoms [1]. STN open-loop, continuous deep brain stimulation (cDBS) effectively alleviates motor symptoms but does not respond to dynamic fluctuations in pathological neural activity over time. Recent advancements in DBS systems have enabled local field potentials sensing and tracking of relevant neural biomarkers. Embedded control policy algorithms use these to adjust stimulation parameters in real time, known as adaptive deep brain stimulation (aDBS). Emerging research on aDBS has demonstrated safety and feasibility in operating room and laboratory settings, and suggested potential for superior therapeutic benefits over cDBS [2–4]. However, research on aDBS in long term at home environment is still limited, primarily focusing on tremor and dyskinesia and often relying on patient self-reports [5–7].

In this study, an individual with clinically established PD underwent long term beta power-driven aDBS at home with a commercially available sensing neurostimulator (Percept^™^ PC, 3389 legacy leads, Medtronic, Inc.), whose aDBS firmware had been unlocked for investigative purposes. The outcome of aDBS was evaluated against their optimized clinical cDBS settings by a remote daily mobility task using Quantitative DigitoGraphy (QDG), validated for quantitative metrics of cardinal motor signs in PD [8]. The participant gave informed written consent approved by the Stanford Institutional Review Board.

The participant was a male patient in his sixties with 13-year of PD history, who had been receiving bilateral STN-DBS for 12 years. Due to medication-induced dyskinesia, he was not on dopaminergic medication and relied solely on DBS therapy, which was optimized at 130 Hz. At study enrollment, he presented with a total score of 43 on the Movement Disorder Society Unified Parkinson’s Disease Rating Scale (MDS-UPDRS) III, with a gait sub-score of 2 while off therapy.

Before aDBS programming, the Percept^™^ signal test, along with lead reconstruction via imaging, was used to determine the optimal input signals for driving aDBS. The LFP power spectral density (PSD) diagram revealed a prominent LFP power peak (8.4 μVp) around 16.6 Hz in the LSTN, and a small peak (2.8 μVp) around 19.5 Hz in the RSTN; the threshold power to enable aDBS was 1.2 μVp. Lead reconstruction showed that the LSTN lead was optimally positioned within the sensorimotor region, while the RSTN lead was located slightly anterior to the STN (Fig. 1A), which supported the smaller power beta peak detected from that side.

Therefore, we programed unilateral aDBS for the LSTN, keeping the RSTN on cDBS. Calibration was conducted in-lab with beta power (5Hz band) streaming while the participant performed gait tasks and QDG mobility tasks. A therapeutic window of 2.2–2.9 mA (participant clinical intensity: 2.8 mA) was established. A moderate ramp rate (0.05 mA/sec up, 0.025 mA/sec down) was implemented with a dual-threshold control policy to enable aDBS to respond to fluctuations in beta power without causing adverse sensations (e.g. dizziness or parethesias) (Fig. 1B).

After aDBS calibration, the participant entered a blinded at-home phase, where he was randomized to four weeks of aDBS followed by four weeks of cDBS. Beta power was tracked with a 10-min average over 24 hours daily (Fig. 1C). Each afternoon, the participant was asked to perform a 30-s QDG mobility task with each hand, which entails alternately pressing and releasing tensioned levers with the index and middle fingers as fast and regularly as possible [8].

Our findings indicate that LSTN aDBS was safe, feasible and well tolerated for long-term use at home. MDS-UPDRS assessed once after four weeks showed similar motor symptoms ratings between aDBS and cDBS (MDS-UPDRS II/III and PDQ-39 scores are listed in Supplementary Table S1). The participant demonstrated high compliance with remote QDG daily testing, completing 25 tests over four weeks during both aDBS and cDBS (Fig. 1D and E). Right-hand QDG Mobility Score was slightly higher during aDBS (81.2 ± 12.6 vs. 76.4 ± 10.2), with a more stable trend over days, whereas a slight but significant decline (i.e., worsening) was noted during cDBS (*β* = −0.51, *p* = 0.03, Fig. 1D). Additionally, QDG tremor severity and percent time freezing (%TF) were less prominent with aDBS: tremor occurred in 7.4 % of tests on aDBS versus 28 % on cDBS; average %TF was 3.4 ± 3.7 % during aDBS compared to 3.9 ± 3.6 % during cDBS (Fig. 1E). Full metrics are provided in Supplementary Fig. S1. Left-hand performance was unchanged, as the RSTN was on the same cDBS settings. Overall, at-home QDG provided a continuous view of symptom fluctuations, in contrast to the limited ‘snapshot’ assessments in clinic visits, underscoring the feasibility of remote symptom monitoring for PD to optimize therapy through more frequent, at-home evaluations.

Beta power demonstrated a diurnal cycle, being lower overnight (when the participant was presumably asleep) than during the daytime (Fig. 1C–F), consistent with previously reported data from long-term STN-LFP recordings in PD [9]. aDBS correspondingly adjusted amplitude in similar temporal patterns, with average amplitude higher during the daytime (2.6 ± 0.3 mA; 9am-11pm) than during nighttime hours (2.3 ± 0.2 mA; 11pm-9am) (Fig. 1C).

Comparing aDBS and cDBS conditions, during the daytime, beta power was greater during aDBS than during cDBS (Fig. 1F). This increase is likely due to the lower of average stimulation amplitude with aDBS (approximately 7.5 % reduction compared to cDBS), thereby a lower total electrical energy delivered (TEED). During the nighttime, beta power remained comparable between the two conditions (Fig. 1F), despite aDBS reducing stimulation to the minimum amplitude limit (2.2 mA) overnight. As the goal of the present study was to compare aDBS with the clinically optimized cDBS settings, we did not adjust the cDBS amplitude to match TEED across conditions. Overall, our finding is consistent with previous studies demonstrating that aDBS achieves similar efficacy to cDBS despite reduced TEED [2,3,5].

This study is the first to implement comprehensive at-home symptom monitoring for long-term fully embedded aDBS for PD. Beta-driven aDBS at home was safe, well tolerated, and daily QDG monitoring suggested improved efficacy compared to clinically optimized cDBS, which showed a significant decline of the Mobility Score over the four-week period. These data provide insight that patients who have clinically optimized cDBS for long term can have substantial day-to-day variation in their motor symptoms. The MDS-UPDRS III tested once after 4 weeks of aDBS or cDBS was not different. While this participant was off medication throughout the study, similar aDBS control algorithms are applicable for people with PD on medication, as supported by prior work including the ADAPT-PD trial [7,10]. Our findings establish proof of principle for continuous remote neural and behavioral monitoring to measure more precisely the real-world efficacy of aDBS, paving the way for future remote optimization of aDBS, guided by long term at-home data.

## Supplementary Material

Supplementary Material

## Figures and Tables

**Fig. 1.**
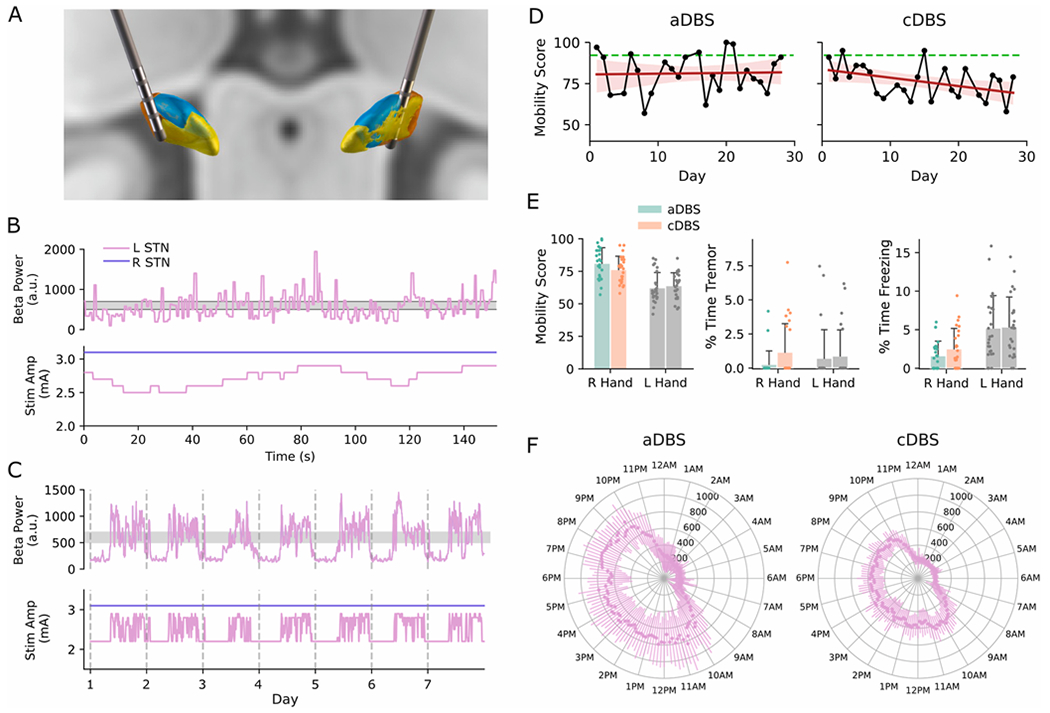
(A) Lead locations in the LSTN and RSTN (orange: sensorimotor; yellow: limbic; blue: associative). (B) In-lab aDBS calibration with real-time streaming of LSTN beta power relative to the dual thresholds during gait. Stimulation amplitude for LSTN decreased when beta power fell below the lower threshold and increased when beta power exceeded the upper threshold; Stimulation amplitude for RSTN remained constant over time. (C) At-home recording of LSTN beta power and stimulation amplitude (10-min averages) during aDBS, shown over seven days. (D) Right hand QDG Mobility Scores during the aDBS and cDBS phases, with linear regression lines (red) and corresponding 95 % confidence interval. aDBS showed no significant linear change (β = 0.04, *p* = 0.88, R^2^ = 0.001), while cDBS had modest but significant decline (β = −0.51, *p* = 0.03, R^2^ = 0.18). Green dash line marks the ‘healthy’ threshold (E) Bar plots of QDG motor symptom metrics during aDBS and cDBS phases; error bars represent standard deviation. (F) Circadian plot of LSTN beta power comparing aDBS and cDBS phases; error bars represent standard deviation over the four-week period.

## Appendix A. Supplementary data

Supplementary data to this article can be found online at https://doi.org/10.1016/j.brs.2026.103028.
