# Supplementary material for: At home monitoring of chronic adaptive deep brain stimulation for Parkinson’s disease
Source: Brain Stimul. Author manuscript; Available in PMC 2026 May 28. (PMC13218403; doi:10.1016/j.brs.2026.103028)
Supplement: Supplementary Material [file NIHMS2173770-supplement-Supplementary_Material.docx]

**Supplementary data: At home monitoring of chronic adaptive deep brain stimulation for Parkinson’s disease**

**Table S1.** Patient-Reported Daily Living, Quality of Life, and MDS-UPDRS III Scores After Four Weeks in Each Condition.

|  | aDBS | cDBS |
| --- | --- | --- |
| PDQ-39 Total Score | 18 | 22 |
| MDS-UPDRS II Total Score | 10 | 9 |
| MDS-UPDRS III Right Body | 12 | 11 |
| MDS-UPDRS III Left Body | 14 | 13 |
| MDS-UPDRS III Total Score | 36 | 32 |

**Figure S1.** Comprehensive QDG Mobility Task Results

**
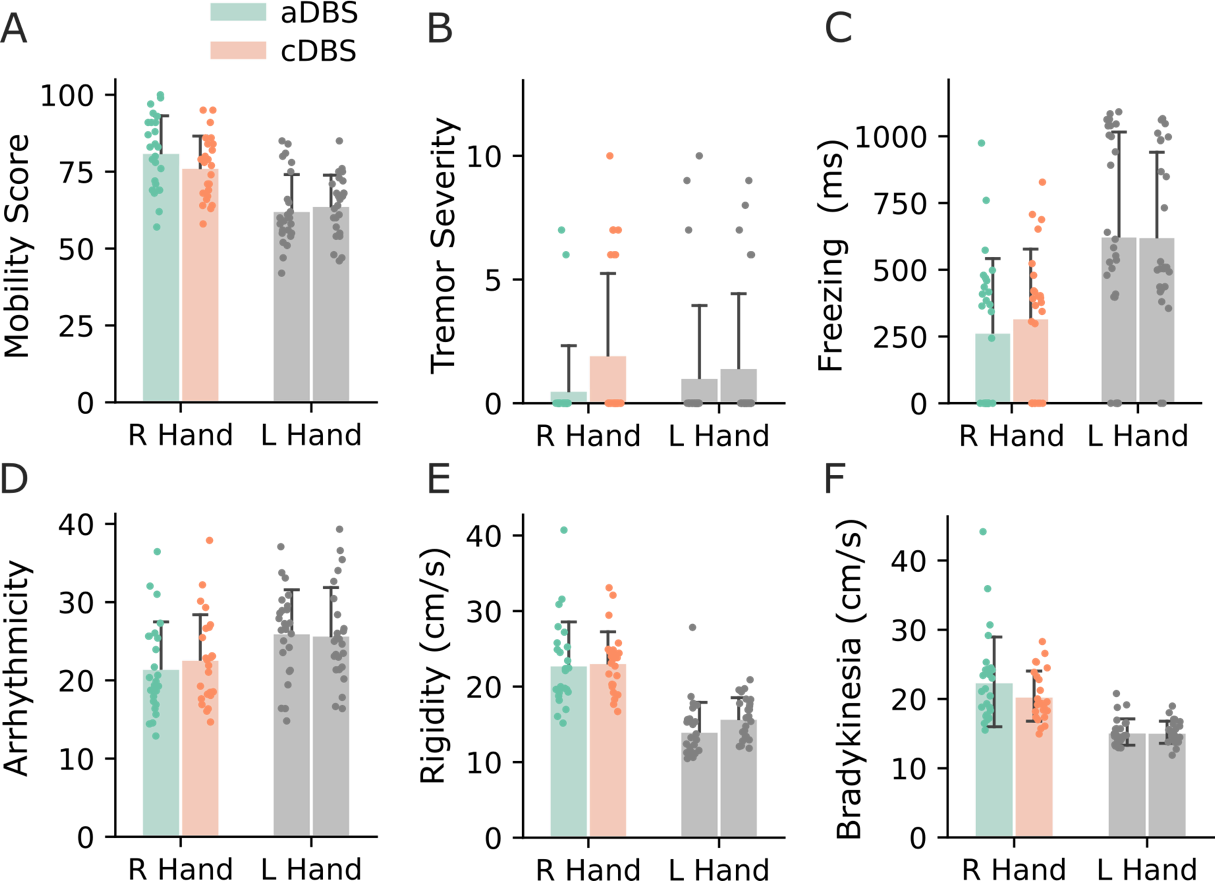
**

(A-F) Bar plots for QDG task metric during aDBS and cDBS phases. Error bars represent standard deviation. Scatters represent scores at each daily test. (B) Tremor occurred in 7.4% of tests during aDBS versus in 28% during cDBS. (C) Freezing during pressing occurred in 55.6% of tests compared with 68% with cDBS.
